# Are we choosing the right flagships? The bird species and traits Australians find most attractive

**DOI:** 10.1371/journal.pone.0199253

**Published:** 2018-06-26

**Authors:** Stephen T. Garnett, Gillian B. Ainsworth, Kerstin K. Zander

**Affiliations:** 1 Research Institute for the Environment and Livelihoods, Charles Darwin University, Darwin, NT, Australia; 2 Coastal Seas Ecology Team, Centre for Ecology and Hydrology, Penicuik, Midlothian, United Kingdom; 3 Northern Institute, Charles Darwin University, Darwin, NT, Australia; Charles University, CZECH REPUBLIC

## Abstract

Understanding what people like about birds can help target advocacy for bird conservation. However, testing preferences for characteristics of birds is methodologically challenging, with bias difficult to avoid. In this paper we test whether preferred characteristics of birds in general are shared by the individual bird species the same people nominate as being those they consider most attractive. We then compare these results with the birds which appear most frequently in the imagery of conservation advocates. Based on a choice model completed by 638 general public respondents from around Australia, we found a preference for small colourful birds with a melodious call. However, when the same people were asked which five birds they found most attractive, 48% named no more than three, mostly large well-known species. Images displayed by a leading Australian bird conservation organisation also favoured large colourful species. The choice model results suggest conservation advocates can promote a much wider range of bird types as flagships, particularly smaller species that might otherwise be neglected.

## Introduction

Birds are often used as flagship species to obtain support for conservation, including public relations, education and fundraising [1–5]. Originally flagship species were conceptualised as “popular, charismatic species that serve as symbols and rallying points to stimulate conservation awareness and action” [6] and “species that have the ability to capture the imagination of the public and induce people to support conservation action and/or to donate funds” [7,8]. However, the definition of flagship species has now evolved to “a species used as the focus of a broader conservation marketing campaign based on its possession of one or more traits that appeal to the target audience” [9]. The modern definition gives greater recognition of the cultural specificity of flagships [1, 10] and of the need to tailor them to the market to which they are trying to appeal [4, 5, 9]. Such tailoring requires knowledge of market preferences. Inaccurate knowledge of preferences can mean that significant segments of the conservation market are overlooked. This means that benefits may accrue to a narrow range of conventional flagship species at the expense of others that may have attracted public support had market research been adequate [11].

For birds, colour, size and shape are often thought to be important in determining human preferences [12]. There are, however, substantial if largely unrecognised methodological difficulties in distinguishing the attractive features of ‘birds’ as a general concept from the attractiveness of particular species of bird. This is because any sample of particular bird species will be complicated by the ways in which those birds have already been encountered by those scoring attractiveness, either as live birds or through cultural representations [10]. Many birds have either implicit or explicit symbolic or totemic values in society [13], with meanings of the same bird types varying among cultures [14]. Social constructs around birds are incorporated into value systems used to create meaning, with preferences potentially established in very young children [15]. The constructs for some species are then reinforced by advertisers and others who link product value to the symbolism associated with particular bird species [16, 17], although there will be others that sustain a value to individuals or cultural groups regardless of their co-option by the market.

This means that preferences derived from images of actual birds may be confounded by cultural preconceptions related to the meanings of those birds–totemic associations that may not be transferable to birds of similar type. As noted by others [18, 19], many programs advocating conservation employ a narrow range of flagship species selected on the basis of preconceptions about the types of species to which the public is likely to respond. However, as with any marketing, product familiarity is critical to consumer preference [20] and any research on understanding preferences must be undertaken in a way that acknowledges levels of knowledge and prior experience. This presents a problem for all studies on preferences for birds to date as they have relied on paintings or photographs of individual bird species [3, 4, 12, 21–24] although some [22, 24] tried to standardise images to minimise bias and, in one study, silhouettes were presented rather than coloured images [22]. Judgements on what is attractive also tend to have been made by small subsets of society such as students [3, 25]; aviculturists [26, 27], book picture editors [28] or self-selected respondents to an internet invitation [22, 24], rather than a more random sample of the general public. Also selective are the number of webpages returned by a targeted web search based on bird name [12, 23] or an index derived from hunting, scientific and natural history literature [21]. This is not to say that the conclusions drawn from this research are incorrect but rather that flagship selection currently rests on still untested assumptions that preferences for an idealised idea of a bird among the general public can be generalised from the preferences for particular named and illustrated birds among self-selected or small subsets of the wider population.

The purpose of the current paper is to refine understanding of the preferences of the Australian public for birds with the aim of improving flagship choice in conservation advocacy [29]. Our principal research question was whether the public’s preference for birds as a theoretical concept differs from what they might articulate when given a choice of particular birds. Even though our results may be used for raising funds for bird conservation, we did not attempt to put a market value on particular birds, as that would have engaged our respondents in the question of whether natural entities all have a monetary value. Rather we sought simply to explore aesthetic preferences to test the evidence base for selection of flagship species. By asking a sample of the general public to reveal preferences for birds without anchoring their potential choices to images of species to which they may already have attachment, we wanted to test the hypotheses that the preferences of the general public for birds may differ from those commonly used as flagship species. We think the results of this research can improve the evidence base for strategies that aim to align bird conservation appeals to human aesthetic preferences for birds.

## Materials and methods

To test our hypothesis, we employ a choice model to explore which aesthetic and other features of birds members of the Australian public find most attractive, avoiding imagery that may anchor respondents to particular species that may have public salience. We then compare this to a list of species the same people say they find most attractive, and the reasons for their selection. We also analyse the characteristics of the birds depicted on the websites of an Australian bird conservation group (BirdLife Australia and its associated organisations) and with the results of a plebiscite among 52 bird species undertaken by BirdLife Australia among its members and their social networks.

### Data collection

We undertook an online survey in February 2011 using a research company (MyOpinions Pty Ltd). This company had, at that time, an active panel of 300,000 verified respondents drawn from the general public (1.2% of Australian population) who were recruited via television, radio, newspaper, and online (without having received any payment). The research company sent out email invitation with the link to our survey to a random sub-sample of their panel. This invitation only contained information about the time needs to complete the survey and the size of the incentive (respondents were paid the equivalent of US$3 on completion). No information about the topic of the survey was revealed in the invitation to avoid decisions to participate based on the research topic. Those people who decided to participate must have followed the link to the first page of the survey which contained information about the research scope and aims, and the research team. This first page also stated that the survey was voluntary and that participants were free to stop any time. It also contained an ethics statement and contact information should respondents have wanted to make comments or complaints. By proceeding from the first page to the first question respondents gave consent. The collected data were non-identifiable. We obtained approval to collect data from the Charles Darwin University Human Research Ethics Committee (H11059).

We opted for an online survey because they are cost-effective and data can be collected in a short time frame. The data from preference surveys obtained through online surveys have been compared to data from other survey modes and found to yield no differences in preferences of willingness-to-pay estimates [30–34]. However, some caution is needed from survey mode effect when undertaking online surveys. Sample characteristics can differ across different modes. For example, respondents to online surveys are often better educated, younger and having higher incomes [31, 33, 34] than those from mail or in-person surveys. Attitudinal characteristics can also differ between online and other modes [33, 34].

### Sampling and response rate

The research company randomly sampled 5,800 people from the panel. Sampling was done to match the characteristics of the panellists to those of the sampled population, which were derived from the national census [35]. This means the randomly selected sub-sample of the panel to whom the research company sent the invitations had an equal number of men and women and an age distribution similar to that of Australia as a whole (except that all respondents were over 18 years old).

Of the 5,800 panellists, 1,229 people agreed to undertake the survey and followed the link. Of these 1,229 people, 470 people (38%) dropped out before completing, including 397 (32%) who dropped out during the first choice sets. We therefore obtained 759 responses out of which we had to discard responses from 121 people because they had not answered the key questions in sufficient detail. The overall response rate of 11% (638/5,800) was not unexpected as response rates of online surveys are usually lower than in surveys of other modes [36–38]. The low response rates for online surveys might be because the benefits from the ‘feeling of importance’ when participating in a survey are lower in online surveys due to lack of personal contact with an interviewer or because the barrier not to participate is lower due to this missing personal contact [33]. The response rate of 52% among those who opened the survey so knew its contents (638/1,229) is a better representation of the response rate and is higher than most postal or telephone survey returns [36–38].

### Questionnaire

The survey consisted of (i) basic demographic questions (gender, age, location), (ii) attitudinal questions about attitudes to birds and ability to identify them, (iii) a choice experiment to explore the characteristics of birds that people find most attractive, (iv) questions asking people which particular birds they find most attractive, and (v) sociodemographic questions (see S1 Appendix for a copy of the questionnaire). We allowed respondents to define ‘attractiveness’ as they saw fit at the time of completing the questionnaire as we wished to capture as many of the physical, emotional, behavioural and cognitive attributes of the term as they wished to apply. Additionally, we asked respondents about their attitudes towards birds (Likert scale questions; see S1 and S2 Figs), about their level of bird knowledge, their socio-economic background (gender, age, income, education level and location), membership of nature-based organisations and participation in nature-based activities, all factors we considered likely, on the basis of the literature, to affect opinions about the attractiveness of birds.

For the most attractive bird elicitation, respondents were asked:

‘Thinking about the different bird species found around Australia, which do you think are the most attractive overall? Why do you find these birds the most attractive? (Please tell us about up to five birds and provide their full names if you can).’

### The choice experimental design

Discrete choice experiments ask survey respondents to make choices, choosing the single option they most prefer from a number of possible options. Each alternative profile is defined by its attributes and each attribute can take on a number of levels. In this survey, respondents were asked to choose between three hypothetical birds defined by a combination of five attributes. These attributes and their levels were finalized after literature review, consultation with experts (3 biologists, 2 economists), and a trial of the choice sets with 10 non-biologist respondents in Darwin in northern Australia. Each choice set was made up of three hypothetical birds (alternatives) from which to choose. The choice sets were unlabelled, i.e. the choice questions referred to bird A, B and C, and described their characteristics but did not provide actual bird names. A stylized set of general silhouettes was provided in the header to each choice set as guidance on size. We assigned three levels to each of the five attributes (Table 1). The total number of possible combinations of all attributes and levels, and therefore the number of possible bird choice sets, were 243 (3^5). We therefore created a manageable number of subsets of all possible combinations. We used the software package Ngene to design a statistically efficient subset based on D-optimality. D-efficiency is a very common measure to determine an optimal experimental design [39]. The D-efficiency is a measure of a design’s ‘efficiency’ relative to the design with the lowest D-error. The final design had a D error of 0.194. The design yielded 12 choice sets which were, using the same software, divided into four blocks. Each respondent was given one block with three choice sets to answer.

**Table 1:** Attributes and levels used in the choice experiment to characterise the bird profiles.

Attribute	N Levels	Levels		
Appearance	3	Colourful	Boldly-marked	Grey-brown
Size	3	Large	Medium	Small
Song	3	Melodious	Quiet	Harsh
Behaviour	3	Confiding	Spectacular	Secretive
Status	3	In danger of extinction	Rare but not threatened	Common

Our design did not include a monetary attribute, i.e. did not ask people to trade off the costs with an attribute, but instead assumed that individuals would select the alternative with the highest levels of the attributes that might make a bird attractive to them while minimising the levels of the attributes that might make a bird unattractive to them. Choice designs without a monetary attribute have been used elsewhere when the aim of the research was not, like in our case, to estimate respondents’ willingness-to-pay [4, 40, 41]. Often the reason for not estimating a willingness-to-pay is a moral or ethical one [40, 42] or the assumption that respondents will be unable or will refuse to make the necessary trade-offs [43, 44] and their uncertainty in making a decision about the value for a public good, even when information is provided [44].

### Analysis

#### Choice experiment analysis to assess bird traits

Choice experiments have become a common method in marketing, transportation and tourism research and health economics, even among sample populations with little knowledge of the experimental subject. They are also common in environmental economics where they are commonly employed to place a dollar value on environmental and public goods. The method has been widely applied in wildlife [45, 46] and domestic animal conservation [47, 48]. Recently they have been applied to explore preferences among flagship species [3–5, 19], including birds [49, 50].

We estimated a latent class (LC) model to account for preference variation across respondents [51] detailed model specifications. More specifically, with the LC model we can identify the characteristics of the segments of respondents that value certain bird traits the most. We used BIC as our criterion for model selection. The reason we opted to use BIC over AIC is that AIC usually overestimates the number of classes, so selects overly complex models [52].

#### Calculation of attractiveness scores

Using the coefficients obtained from the choice experiment, and following the assignment of characteristics based on the established criteria (Table 2) an attractiveness score (A) for bird *i* was computed following the approach suggested by [53]. A list of all attractiveness scores is provided in the data repository associated with this paper.

$$A_{i}=x_{i}\hat{\beta }$$ (1)

where *i* denotes the bird of interest and $\hat{\beta }$ is the vector of coefficients derived from the choice experiment.

**Table 2:** Criteria for characterising birds used on Birdlife Australia websites in order to assess their attractiveness.

Attribute	Level	Decision criteria	Examples of Australian birds in this category
Appearance	Colourful	Large patches of bright colour	Rainbow Lorikeet, Rainbow Bee-eater, Scarlet Robin
	Boldly-marked	Strongly contrasting patches of plumage	Red-kneed Dotterel, Black Honeyeater, Willie Wagtail,
	Grey-brown	Neither much bright colour nor strong patterning	Brown Falcon, Dusky Robin, Little Woodswallow
Size	Large	Wt (g) x Length (cm) >1,000,000 [76]	Eclectus Parrot (smallest), Eastern Curlew (median), Ostrich (largest)
	Medium	Wt (g) x Length (cm) >70,000 and ≤1,000,000 [76]	Common Myna (smallest), Common Greenshank (median), Buller's Shearwater (largest)
	Small	Wt (g) x Length (cm) ≤70,000 [76]	Weebill (smallest), Black-winged Monarch (median), Little Wattlebird (largest)
Song	Melodious	Call described in positive terms in HANZAB [76]	Black Swan, Australian Magpie, Flame Robin
	Quiet	Calls described as being usually quiet in HANZAB [76]	Southern Emu-wren, Double-barred Finch
	Harsh	Call described in negative terms in HANZAB [76]	Australian White Ibis, Rainbow Lorikeet, Spangled Drongo
Behaviour	Confiding	Readily approachable. Assessed subjectively based on flight distance [54]	Bar-shouldered Dove, Dusky Moorhen, White-plumed Honeyeater
	Spectacular	Particularly aerial displays. Assessed subjectively based on descriptions of behaviours in HANZAB [76] and personal experience of assessors	White-throated Needletail, Peregrine Falcon, Dollarbird
	Secretive	Rarely seen in the open. Assessed subjectively based on descriptions of behaviours in HANZAB [76] and personal experience of assessors	Brown Quail, Blue-billed Duck, Pilotbird
Status	In danger of extinction	Listed as threatened or Near Threatened in [54]	Southern Cassowary, Fairy Tern, Regent Parrot
	Rare but not threatened	Not in danger of extinction but occurring in ≤5 Interim Bioregionalisation of Australia Bioregions (out of 85) or with a reporting rate (no. records/no. lists) in first and second Australian bird atlases of ≤5 [77]	Little Ringed Plover, Rufous Owl, Little Kingfisher
	Common	Neither of the above	Australian Wood Duck, Glossy Ibis, Black Kite

#### Analysis of the most attractive birds

Allowing participants to nominate species of their own rather than using a pre-selected list provided an opportunity to explore breadth of knowledge about bird taxa within the cross-section of Australians sampled and their preferences. Responses regarding respondents’ favourite birds were analysed to identify each bird nominated to the level of order, family, genus and species, using the taxonomic base of [54]. In addition attractiveness scores were calculated for each bird chosen and mean values for classes identified by the CL model compared. All scores were also compared with the average for all Australian birds. In addition we scored all birds against the three levels of each of the five attributes used in the choice model and used Chi-square to compare birds chosen by the latent class groups with the each other and all Australian birds.

#### Analysis of conservation advocacy photos

We searched the websites of all Birdlife Australia branches within Australia for their use of bird pictures (in both 2014 and 2016). Although no web managers were interviewed to determine choice and motivation, all images were portrayed on the page in ways that would appear to have maximised their visual appeal so are effectively acting as flagships for birds as visually appealing objects. The birds depicted on the websites were classified according to the attributes used in the choice experiment. This was done by three independent bird experts who established criteria for the categorisation beforehand (Table 2). All birds used by Birdlife Australia branches were then scored according to the preferences respondents assigned to the characteristics and an attractiveness score was calculated for each of the birds from the results of the choice experiment (Eq 1). Low scores signify a low attractiveness score by the public, a high score a high attractiveness. The reason we used the birds shown on the Birdlife websites was to have some representative examples of birds used for conservation advocacy. As with the selections of most attractive birds from our survey, attractiveness scores for species chosen by BirdLife Australia were compared with those for all Australian birds occurring regularly on the Australian mainland or in Tasmania.

Additionally, we compared the scores for the birds found most attractive among our sample to those named in an online survey of Australia’s birds which was conducted by BirdLife Australia in November 2013 among its 8,000 members and their social networks. In the BirdLife survey, which conducted via a web link on their website where people were asked to choose from a list of 52 species, each with a photo, selected from those recorded most commonly in a recent national bird atlas [55] as well as four threatened species that are the subject of BirdLife Australia projects. The plebiscite was not impartial–people were asked to advocate for their favourite bird–but is the only recent survey related to our own.

## Results

### Sample characteristics

Of the 638 respondents, 59% were female. In accordance with the predetermined sample request, respondents were distributed relatively evenly across all age categories (18–24:11%, 25–34:13%, 35–44:17%, 45–54: 21%, 55–64:17%, 65+:21%). Also by request, the geographical distribution of respondents matched the demographic variation among Australian states (New South Wales 29%, Victoria 25%, Queensland 21%, Western Australia 10%, South Australia 8%, Tasmania 3%, the Australian Capital Territory 2% and the Northern Territory 1%).

In total, 28% of respondents were members of some kind of environmental organisation, with most belonging to a conservation group (15%) or an animal welfare society (12%). Very few respondents belonged to a national (2%) or local (<1%) bird group, so no bias can be detected here. Sixty-four percent of respondents agreed that they can identify common birds in their area (S1 Fig). Over half paid attention to birds wherever they go (58%) and 45% agreed that seeing a new bird fills them with excitement (S2 Fig). About 15% agreed that they paid no attention to birds.

### Traits of attractive birds derived from choice experiment

As a starting point, we ran a conditional logit (CL) model and then proceeded to a latent class (LC) model to shed more light on preference heterogeneity among respondents. The CL model results showed that respondents did not value all levels of bird traits (Table 3). For appearance of the bird, the levels ‘Boldly-marked’ and ‘Grey-brown’, for instance, were not statistically significant from one another, but both were less preferred than ‘Colourful’. For behaviour, ‘Spectacular’ and ‘Secretive’ were not different from each other while ‘Confiding’ is significantly less preferred than these two levels. For the status, only the level ‘In danger of extinction’ was significant and preferred over the other two levels which were not statistically different from each other (‘Rare’ and ‘Common’). Regarding bird size, respondents preferred ‘Small’ over “Medium’ and both over ‘Large’, and regarding song, ‘Melodious’ over ‘Quiet’ and both over ‘Harsh’. The highest ranking of the attributes from the CL model was ‘Small’, followed by ‘Melodious’, ‘Medium size’, ‘Colourful, ‘Endangered’ and ‘Confiding’ with ‘Harsh’ having the lowest rank. Overall, size was the most important trait with respondents being more than twice as likely (OR: 2.23) to choose a small bird profile than a large one and about 70% more likely (OR: 1.65) to choose a medium-sized bird profile than a large one.

**Table 3:** Results of two models estimated with the data obtained from a choice experiment: A conditional logit (CL) and a latent class (LC) model.

	CL model			LC model					
				Class 1 (65%)			Class 2 (35%)		
	Coeff.	SE	Odds ratio	Coeff.	SE	Odds ratio	Coeff.	SE	Odds ratio
Size: Small	0.800***	0.074	2.23	1.944***	0.307	6.99	-0.463	0.298	0.63
Size: Medium	0.499***	0.089	1.65	1.416***	0.264	4.12	-0.336	0.257	0.71
Appearance: Colourful	0.403***	0.082	1.5	0.625***	0.158	1.87	0.198	0.209	1.22
Song: Melodious	0.547***	0.087	1.73	0.806***	0.181	2.24	0.189	0.214	1.21
Song: Harsh	-0.284***	0.106	0.75	-0.155	0.187	0.86	-0.498**	0.254	0.61
Behaviour: Confiding	-0.191***	0.068	0.83	0.018	0.119	1.02	-0.538***	0.185	0.58
Status: Endangered	0.328***	0.067	1.39	0.072	0.131	1.07	0.557***	0.165	1.74
Constant Option A	0.09	0.069		0.180	0.139		0.163	0.120	
Constant Option B	0.098	0.069		0.185	0.132		0.119	0.158	
*Explaining class membership*:									
Constant				0.025	0.483				
Female				0.971***	0.291	2.64			
Birds have a right to exist				0.333**	0.146	1.40			
Birds are a nuisance				-0.346**	0.136	0.71			
BIC	3177.3			3175.0					
Log likelihood	-1559.1			-1509.9					
Pseudo R-squared	0.12			0.15					

***, ** = significance at 1%, 5%

A 2-class LC model minimized Bayesian Information Criteria (BIC) and was chosen as the final choice model (Tables 3 and 4). The majority of respondents (65%) belonged to class 1 and they looked at the size, song and appearance of the bird profiles when making their choices. Those in class 2 (35%) looked at the status, song and behaviour. Respondents in class 1 liked colourful small or medium birds with a melodious song and those in class 2 opted for endangered birds that did not have a harsh sound or confiding behaviour.

**Table 4:** Criteria for determining the optimal number of classes (for LC models without covariates).

Classes	Number of variables	Log-Likelihood	BIC
2	19	-1521.1	**3182.6**
3	29	-1501.3	3216.9
4	35	-1484.8	3228.2
5	49	-1467.5	3297.1

We also explored whether some factors affect the membership of the classes. Therefore we added individual characteristics of the respondents, and found that gender and two of the attitudes had significant impacts on class membership. Women had higher odds of being in class 1 than in class 2 (more than twice that of men, OR: 2.64), as had those who thought that ‘the bird has a right to live only if it is beautiful or unusual’. Those who felt that ‘it’s a nuisance when an endangered bird stops development’ were less likely to belong to class 1 than to class 2.

### Attractive birds nominated by respondents

Over 92% (584) of the 638 respondents named at least one bird and around half (311) named five birds; an average of three birds were named per respondent. In total, 2173 nominations were made from a possible 3190 and a total of 198 unique bird taxa were nominated, usually by common name. Respondents demonstrated great variability in their knowledge of bird names, and nomenclature accuracy varied widely. Overall, 461 of the respondents identified at least one bird identifiable to species (e.g. Tawny Frogmouth *Podargus strigoides*) although half of these (220) included Laughing Kookaburra *Dacelo novaeguineae* or Australian Magpie *Cracticus tibicen* within their five and only 56% of nominations (1212 votes) were identifiable to species level overall; a further 27% of nominations (592) were identifiable to genus level (e.g. fantail); 17% of nominations (365) could only be recognized at family level (e.g. ‘birds of prey’); and just three nominations could only be recognized at the level of order or less (e.g. ‘night birds’). Misspelling of names was common. Just 22% were spelled according to the standards set by BirdLife Australia [54]. Although some misspellings could be accounted for by typographical errors, there were also several literal renderings of names pronounced with an Australian accent (e.g. *glar* (Galah *Eolophus rosiecapillus*), *laracetes* (Lorikeets *Trichoglossus* spp.), *cocabara* (Kookaburra)).

The diversity of birds nominated in the survey represented 18 of the 22 orders of Australian native birds, 54 of the 102 families, 105 of the 369 genera and 108 of the 720 species currently recognised [54] as well as including three families (hummingbirds, toucan, condors) not occurring in Australia.

Birds from the order Psittaciformes (parrot and cockatoos) were most popular overall with 849 nominations allocated between two families: Psittacidae (66% of votes for this order) and Cacatuidae (34%); overall 78% of respondents naming birds selected at least one parrot or cockatoo. The most commonly represented taxa from this order, identifiable to species level, were Rainbow Lorikeet *Trichoglossus haematodus* (Psittacidae) with 89 mentions (4% of all nominations) followed by Galah (Cacatuidae) with 62 (3%).

The second most frequently represented order was Passeriformes (perching or song birds) with 625 nominations. Artamidae was the most popular family with 185 votes (30% of votes for this order) and the Australian Magpie received most votes of all identifiable species in this family (156, 7% of all nominations). An additional 82 votes (4%) were cast for Willie Wagtail *Rhipidura leucophrys*, the only species identifiable from the Rhipiduridae.

**Fig 1:**
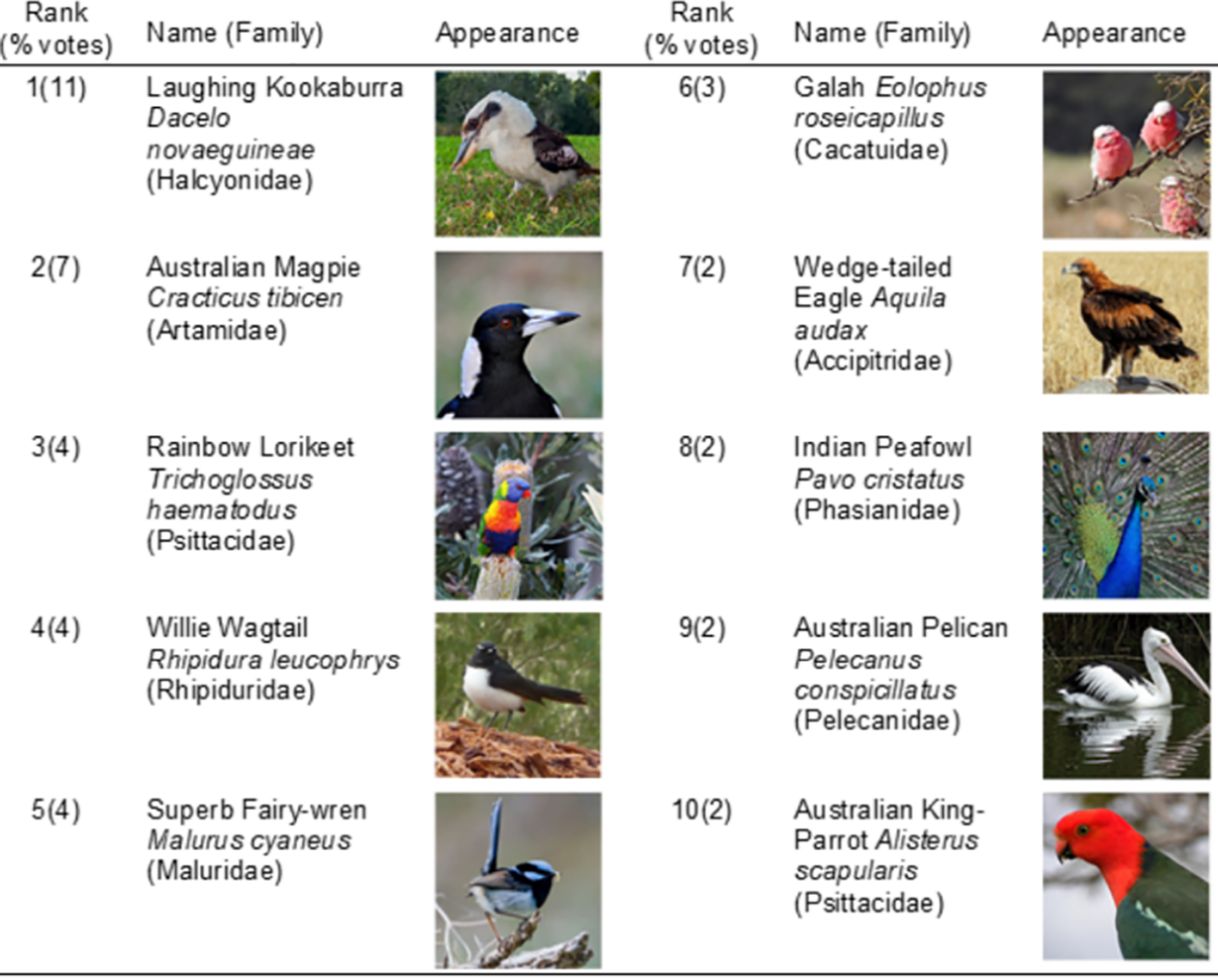
Top ten Australian bird species as nominated by survey respondents as the most attractive ranked in order of number of nominations received (n = 2,173 votes).

The third most commonly represented order was Coraciiformes (near passerines or arboreal birds) which received 267 votes. The majority of these votes were for the Laughing Kookaburra, of the family Halcyonidae (242 votes, 11% of all nominations). The ten most frequently nominated bird species are listed in order of number of votes in Table 5; 51 (%) of taxa received just one vote. The populations of all ten species are widely distributed across Australia and listed as “Least Concern’ according to the IUCN Red List criteria [54] (Fig 1).

**Table 5:** Top ten bird species as nominated by survey respondents as the most attractive ranked in order of number of nominations received.

Rank	No of votes (% of all votes)	Common name	Species	Family	Geographic distribution
1st	242 (11%)	Laughing Kookaburra	*Dacelo novaeguineae*	Halcyonidae	National except NT
2nd	156 (7%)	Australian Magpie	*Cracticus tibicen*	Artamidae	National
3rd	89 (4%)	Rainbow Lorikeet	*Trichoglossus haematodus*	Psittacidae	National except NT
4th	82 (4%)	Willie Wagtail	*Rhipidura leucophrys*	Rhipiduridae	National
5th	78 (4%)	Superb Fairy-wren	*Malurus cyaneus*	Maluridae	ACT, NSW, Qld, SA, Tas, Vic
6th	62 (3%)	Galah	*Eolophus roseicapillus*	Cacatuidae	National
7th	41 (2%)	Wedge-tailed Eagle	*Aquila audax*	Accipitridae	National
7th	41 (2%)	Indian Peafowl	*Pavo cristatus*	Phasianidae	National (domestic)
9th	40 (2%)	Australian Pelican	*Pelecanus conspicillatus*	Pelecanidae	National
10th	39 (2%)	Australian King-Parrot	*Alisterus scapularis*	Psittacidae	ACT, NSW, Qld, Vic

ACT = Australian Capital Territory; NSW = New South Wales; NT = Northern Territory; QLD = Queensland; SA = South Australia; Tas = Tasmania; Vic = Victoria; WA = Western Australia.

The mean attractiveness of bird taxa selected by Class 1 people (1.03±st.dev.0.31) did not differ from either the score for Class 2 people (0.98±0.31) and was not significantly lower than the mean score for all birds listed in Australia (1.12±0.54) although 58% of Class 1 birds and 68% Class 2 birds had below average attractiveness scores. Although Class 1 respondents found colourful, melodious, small-medium sized birds the most attractive, 46% of birds chosen were grey-brown or boldly marked but not colourful, 25% were large, and 82% had harsh calls or were usually quiet (Table 6). Class 2 respondents found threatened birds attractive but ostensibly eschewed birds with harsh calls or which are confiding. Nevertheless 38% of the birds chosen have harsh calls and 38% are confiding while just 2% of those selected are threatened with extinction. The proportions of birds in the different call and rarity categories did not differ between classes but Class 1 respondents did tend to choose smaller, more colourful and more confiding birds than respondents in Class 2, in line with their preferences revealed by the latent class model.

**Table 6:** Characteristics of birds selected among the five most attractive according to respondents’ latent class.

Sample	Colourful	Boldly-marked	Grey-brown	Test	Χ^2^	P
Class 1	55	32	14	Class 1 x Class 2	7.3	0.030
Class 2	49	33	17	Birds x Class 1	551.3	<0.001
All birds	22	28	50	Birds x Class 2	410.2	<0.001
	Large	Middle	Small			
Class 1	25	54	21	Class 1 x Class 2	16.9	<0.001
Class 2	33	51	16	Birds x Class 1	245.5	<0.001
All birds	21	26	54	Birds x Class 2	720.0	<0.001
	Melodious	Quiet	Harsh			
Class 1	18	44	38	Class 1 x Class 2	0.7	0.710
Class 2	17	45	38	Birds x sample	8.4	0.020
All birds	17	49	33			
	Confiding	Spectacular	Secretive			
Class 1	44	50	7	Class 1 x Class 2	9.1	0.010
Class 2	38	56	6	Birds x Class 1	804.6	<0.001
All birds	21	21	59	Birds x Class 2	898.6	<0.001
	In danger of extinction	Rare but not threatened	Common			
Class 1	1	2	97	Class 1 x Class 2	1.6	0.460
Class 2	2	2	96	Birds x sample	447.5	<0.001
All birds	12	30	58			

### BirdLife Australia image attractiveness scores

The mean attractiveness score for all 98 bird taxa with images on BirdLife Australia websites was 1.14 (±0.55) which was not significantly different to the mean score of all birds listed in Australia (1.12±0.54)). So overall, the BirdLife Australia list represents a random sample of birds measured by their attractiveness. However, there were significant differences in traits selected by BirdLife Australia compared to all Australian birds. Birds picked for the BirdLife Australia websites were more likely to be more colourful than Australian birds in general (41% vs. 19%; χ^2^ = 22.62, p < 0.001) and less likely to be grey-brown (28% vs. 53%; χ^2^ = 20.38, p < 0.001), and marginally more likely to be large (27% vs. 19%; χ^2^ = 3.07, p < 0.1).

The top 10 birds in the 2013 BirdLife Australia plebiscite had a mean attractiveness score of 1.23 (±0.45) compared to 1.07 (±0.55) for the group of 52 as a whole. Unsurprisingly, given the way in which they were selected, those on the list of 52 were more likely to be common (76% vs. 63%; χ^2^ = 5.96, p < 0.01) and less likely to be rare (8% vs. 24%; χ^2^ = 12.24, p < 0.001). This is consistent with being more likely to show spectacular (28% vs. 19%; χ^2^ = 4.28, p < 0.05) and less likely to show secretive behaviour (47% vs. 61%; χ^2^ = 6.99, p < 0.01). There were no biases for size, song or plumage.

## Discussion

Even though the two methods of assessing the attractiveness of birds, direct nomination and choice modelling, were applied by the same people in the same questionnaire, they produced results that were surprisingly different. The preferences that emerged from the choice model were for species that were colourful, small to medium sized, melodious, confiding and more threatened than the average. However, given the opportunity to name the birds they found most attractive, many of the species people selected are large, with harsh calls and display little colour. In particular only a tiny proportion of the birds selected as a being particularly attractive were threatened or even rare, even though the average attractiveness score for threatened or rare species is no different to that of common species. The difference, we suggest, can be explained by public profile and knowledge of alternatives. Images of all of the top ten species listed by name are commonly depicted on commercial products, stamps and coins; their physical characteristics are celebrated on council logos, faunal emblems and in defense force mascots; and their names are appropriated for place names and sports teams [56–60]. By contrast birds meeting the attractiveness criteria emerging from the choice model have little public profile. We suggest that, just as only a small number of well-known artists dominate prices through selective exposure by galleries and dealers [61], the familiarity and popularity of a few iconic species has created an ‘attractiveness’ feedback loop, effectively excluding many other species that could benefit from having a higher profile.

If corroborated, our findings extend existing concern about the narrowness of the existing flagship fleet. Large mammals and birds tend to be favoured by conservation NGOs as flagship species [18, 62, 63]. Elsewhere [6] it is suggested that this is because such species are easier to detect and distinguish, and thus have higher charisma using the metrics developed by [64]. However, the number of species employed in this way is exceptionally narrow. For example, while the ‘Big five’ dominate African wildlife tourism, at least eight other species elicit an equally strong emotional response from tourists [65]. Similarly other researchers [19] characterised as ‘Cinderella’ species those that share the characteristics of existing flagships but are overlooked. Our findings suggest there is a group of birds that align even more closely with the Cinderella metaphor than the types of animals highlighted in [19] as not only do the small birds miss out on being profiled as flagships, they may actually be considered more attractive were they to be offered as flagships.

We think that there are likely to be two main reasons that people tended to nominate species commonly portrayed in imagery and media. The first is that, when answering the open-ended question on attractiveness, that participants may have been relying simply on familiarity and availability heuristics [66] and, therefore, just named birds that immediately came to mind and be a psychological artifact of human memory [67, 68, 69]. Thus, the two measures of preference may actually tap into different constructs (familiarity versus attractiveness) with only the choice model truly measuring preference. While this could have been the case, this still suggests opportunities for alternative flagship promotion do that it is the birds that people truly like that are front of mind.

The second explanation for the pattern of nominations may be that many respondents simply did not know the names of bird types with the characteristics they actually prefer. This phenomenon may apply to other studies where the sampling method has not provided named examples. For example, web salience of species in Brazil [12] may not reflect popularity but rather familiarity with names. Thus the large and colourful macaws are famous; the names of the smaller species are less well-known so cannot be the subject of web searches. Curiously there is a little evidence that Australians were once more familiar with birds that more closely align with the attractiveness characteristics in the choice model, and so were able to express a preference for them. In 1908 readers of a major Australian metropolitan newspaper were asked to select their “12 best birds” [70]. While nothing is known to the size of the sample, not only did the journalist expect the general readership to be familiar with a wide range of small birds and their names (e.g. flycatchers, robins, tits, native thrush and Welcome Swallow) but the ten most popular species had a score on the attractiveness scale developed from the choice model of 1.46 (±0.51), substantially higher than the national average, the selection by BirdLife Australia or the surveyed public.

If confirmed, this finding has implications for those selecting flagships for advocacy. An assumption that large birds are more attractive than small ones can be interpreted as meaning that the smaller species have a lower value, leading to negative effects on non-flagship species [11]. To some extent this was reflected in our survey. Very few threatened taxa were named among the most attractive species, even though about a third of the respondents fell into a latent class for whom the extent to which a species is threatened was a significant contributor to whether class members thought it attractive. Instead one of the taxa most frequently selected in our survey, the Indian Peacock, is introduced to Australia and is most frequently encountered in captivity. Knowledge that a species is threatened can increase its apparent attractiveness [25] and a willingness to pay for its conservation [71, 72]. In our case we think that, even if people knew of threatened species and found them attractive, they were excluded from attractiveness ratings because people did not know the birds’ names. This lack of knowledge can then influence the choice of flagships aiming to influence the public.

Given that perceptions of attractiveness can be manipulated [25] and that physical attractiveness or charisma is not an essential feature of a flagship [6], the extent to which inherent attractiveness influences flagship effectiveness is moot. Nevertheless, those advocating for bird conservation could usefully test the hypothesis emerging from our results by comparing the response to flagship birds with diverse attractiveness scores. It has long been known that different types of birdwatcher respond to different incentives [73]. This research suggests the broader public may be persuaded to respond to a suite of bird taxa not usually employed to generate support. Indeed it suggests the market may be segmented with women in particular preferring the small colourful melodious species while rarity has a strong influence on the aesthetic choices of another group. In particular our analysis suggests that the general public will be drawn to a far wider range of bird species than is currently presented to them through websites or in Australian bird iconography if they are given the opportunity.

### Limitations and further research

We have identified three aspects of our study that warrant further investigation using other methods. The first relates to the differences in how respondents were asked about their favourite traits (in a choice experiment) and about their favourite birds (as open-ended questions). Open-ended questions are vulnerable to recall bias [74, 75] while choice experiments are limiting the traits that people can choose to define what they consider attractive. Therefore, the divergent results could be partly an artefact of differences between the two methods. Alternatively using the two methods can be viewed positively since it was triangulation between the two methods that revealed the most interesting aspects of the research.

Second we opted for a cost-effective online survey. Like every survey mode, online surveys come with caveats. First an 11% response rate is low. However, this high refusal rate was before potential respondents knew what the survey was about, so was not a reflection of the subject matter. Of more concern is that 48% of those agreeing to undertake the survey failed to complete in a form that could be used. The relatively high dropout rate when faced with the choice sets also suggests that many respondents found them conceptually challenging. Nevertheless, while a meta-analysis has shown that response rates do vary between conventional mail-out and online surveys [37] and that the sample characteristic can differ across survey modes [33, 34], the results of stated preference online surveys have not been found to differ from those using more traditional methods [31–34]. Nevertheless repeating with the work with a different survey technique would test the assumption of similarity for this example.

Third, when our experiment was conducted, relevant work [22, 23] exploring the importance of shape and tone in human preferences for birds, had not been published. Combining choice modelling of a random sample with their questions about shape and colour could be rewarding. Ideally this would be done experimentally in real world situations, such as has been done for mammals [6], so that the hypothetical ideas proposed here are tested empirically.

## Conclusions

We present results from an Australian-wide online survey on the characteristics of birds people found most attractive and also asked them to nominate their top five species. Based on traits alone, respondents preferred birds that are small, colourful, melodious and threatened. However, there was a poor match between these traits and the birds they named directly as being most attractive. Many of the birds people could name are large and iconic, reflecting an exposure in popular imagery. Advocates commonly use birds resembling these iconic species as flagships, potentially to the detriment of those not selected. We suggest that greater resonance with the public, and a greater return on investment in flagship promotion, could be achieved by increasing their exposure to species more closely resembling the types of birds the public actually do find attractive but simply don’t know well enough to name.

## Supporting information

S1 Appendix Questionnaire. (PDF)

S1 Fig Respondents’ ranking (%) of their ability to identify birds by sight and/or sound for different groups of birds—common birds, moderately difficult, difficult and vagrant birds (potential answers were: can identify all birds within a group, most birds, some birds and no birds). (TIF)

S2 Fig Responses (%) to “Thinking about your daily life, how much do you agree or disagree with these statements?”. (TIF)
