# Supplementary material for: Are we choosing the right flagships? The bird species and traits Australians find most attractive
Source: PLoS One. 2018 Jun 26;13(6):e0199253. doi: 10.1371/journal.pone.0199253 (PMC6019765; doi:10.1371/journal.pone.0199253)
Supplement: S1 Appendix — (PDF) [file pone.0199253.s001.pdf]

Supporting Information

S1 Table. Questionnaire

Social Values of Australian Threatened Birds

qualtrics.com

myopinions

Hello from MyOpinions

Thank you for agreeing to participate in this survey. To begin the survey, click on the button below. As you move through the survey please do not use your browser buttons - use the buttons at the bottom of each screen.

Please remember:
- Your views are important to us and your answers will be kept in the strictest confidence.
- None of the responses you give are directly linked to you as an individual. They are used purely for statistical purposes only.
- The survey incentives and expected length are outlined in the invitation e-mail.
- In order for us to reward you for your time and opinion, please complete this survey in one unless specified otherwise.

Honest and thoughtful answers to this survey are vital to the integrity of the market research process. We, and our clients, require factual information in order to make important decisions that not only affect consumers like you; but other people as well.

Please click next if you agree to spend a reasonable amount of time completing this survey and to provide honest and thoughtful responses.

Next

0% 100%

Privacy Policy | Technical Problems? Contact Us

Survey Powered By Qualtrics

Dear participant,

Birds are part of our everyday lives. We share our green spaces with them; we create artworks and write stories about them; we are inspired by their beauty and song. Some birds are good to eat while others can be frightening or annoying.

This survey aims to find out what birds mean to Australians. It is part of a larger PhD study called The Social Values of Australian Threatened Birds which aims to improve the ways we conserve birds and their habitats.

Since this is a public opinion survey, you don't have to be knowledgeable about birds, as the questions cover general topics relating to them. Participation is voluntary. Even if you decide not to participate in this survey, you are welcome to see the results, which will be available on my website later in 2011

Thank you and enjoy the survey!
Gill Ainsworth (Ms)
School for Environmental Research, Charles Darwin University, Darwin, NT, Australia

T. +61 8 8946 7762 | E. gill.ainsworth@cdu.edu.au | W. www.cdu.edu.au

This research is supported by the Birds Australia Stuart Leslie Bird Research Award 2010

CHARLES DARWIN UNIVERSITY

Birds Australia

Back Next

0% 100%

Section 1: Basic demographic questions

Q1. Are you…

□ Male □ Female

Q2. What was your age last birthday?

□ 18-24	□ 25-34	□ 35-44
□ 45-54	□ 55-64	□ 65+

Q3. Where do you live?

□ ACT	□ SA
□ NSW	□ TAS
□ NT	□ VIC
□ QLD	□ WA

Section 2: Attitudinal questions about attitudes to birds and ability to identify them

Q4. Thinking about your daily life, how much do you agree or disagree with these statements? (tick one option for each statement)

	Strongly disagree	Disagree	Neither Agree nor Disagree	Agree	Strongly Agree
I pay attention to birds wherever I go	O	O	O	O	O
I can identify common birds in my area	O	O	O	O	O
Seeing a new bird fills me with excitement	O	O	O	O	O
I am not really interested in birds	O	O	O	O	O

Q5. Please rate your overall ability to identify birds by sight and/or sound. (tick one option for each statement)

I can identify...

	None	Some	Most	All
Common birds, e.g. magpie, cockatoos, kookaburras	O	O	O	O
Moderately difficult birds e.g. honeyeaters, owls, waterfowl	O	O	O	O
Difficult birds e.g. seabirds, waders, thornbills, corvids	O	O	O	O
Vagrants	O	O	O	O

Q6. Many of Australia's birds are endangered (1 in 5 species) and it's uncommon to see an endangered bird in the wild.

Thinking about how you would feel if you knew you had seen an endangered bird, how much do you agree or disagree with these statements? (tick one option for each statement)

If I saw an endangered bird, I might...

	Strongly disagree	Disagree	Neither Agree nor Disagree	Agree	Strongly Agree
want to learn more about the bird	○	○	○	○	○
add it to my birdwatching list	○	○	○	○	○
regret that humans had caused the bird to become endangered	○	○	○	○	○
think there's a moral obligation to protect the bird	○	○	○	○	○
feel it's a nuisance when an endangered bird stops development	○	○	○	○	○
think the bird has a right to live only if it's beautiful or unusual	○	○	○	○	○
feel the needs of people come before those of endangered birds	○	○	○	○	○
think government is responsible for the bird's survival, not me	○	○	○	○	○
feel upset if the bird became extinct	○	○	○	○	○
feel privileged or spiritually uplifted	○	○	○	○	○

Section 3: Choice experiment to explore the characteristics of birds that people find most attractive

The next three questions ask you to pick your preferred bird from a range of different options.

Each question shows descriptions of three different birds. There is information about size, appearance, call, behaviour and conservation status.

The image is there to help you visualise birds of different shapes and sizes – small (dark shading), medium (mid-shading) and large (light shading)

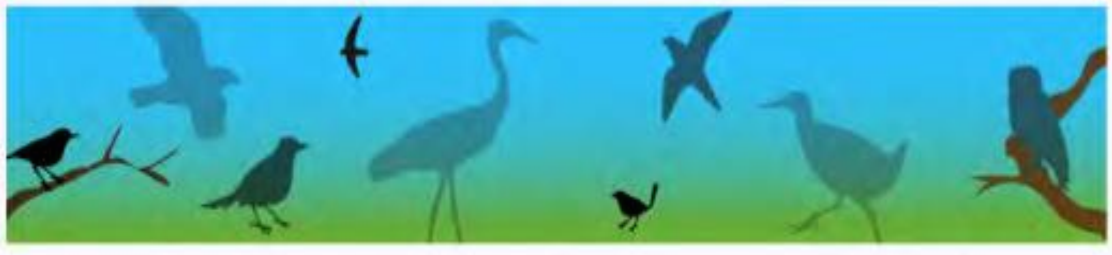

Example of choice set (3 sets given per respondent)

	Bird A	Bird B	Bird C
Size	Large (~ 50 cm)	Medium (~ 15 cm)	Small (~ 8 cm)
Appearance	Subtle brown, black and grey	Bold black, white and grey	Colourful scarlet, green and blue
Call	Sweet-sounding, fluting warble	Quiet with occasional soft chatter	Harsh abrasive crowing
Behaviour	Secretive and rarely seen in open	Confiding and readily approachable	Spectacular aerial display
Conservation status	Rare but widespread, not threatened	Common over extended range	Rare but widespread, not threatened

I prefer: ☐ ☐ ☐

Section 4: Questions asking people which particular birds they find most attractive

Q 7. Thinking about the different bird species found around Australia, which do you think are the most attractive overall?
Why do you find these birds the most attractive?
(please tell us about up to five birds and provide their full names if you can)

	Most attractive birds<br>Name of bird	Why do you say that?<br>Reason for choice
Bird #1		
Bird #2		
Bird #3		
Bird #4		
Bird #5		

Section 5: Sociodemographic questions

Q8. What is the highest level of education you have completed?
□ Year 12 or below
□ Trade certificate
□ Bachelor degree or equivalent
□ Postgraduate degree (MSc, PhD etc.)
□ Other (please specify) ___________________________

Q9. How would you derive your current work situation?
□ Conducting own business
□ A wage or salary earner
□ A helper not receiving wages
□ Other (please specify) _______________
□ A student
□ Homemaker
□ Retired / Semi-retired

Q10. What is your gross income from all sources (including pensions and allowances)?
□ up to $ 40,000 per year
□ $41,000 to $80,000 per year
□ $81,000 or more per year
□ I don’t know / Prefer not to say

Q11. Where were you born?
□ Australia
□ England
□ Scotland
□ New Zealand
□ Italy
□ Greece
□ Viet Nam
□ Other (please specify) ___________________

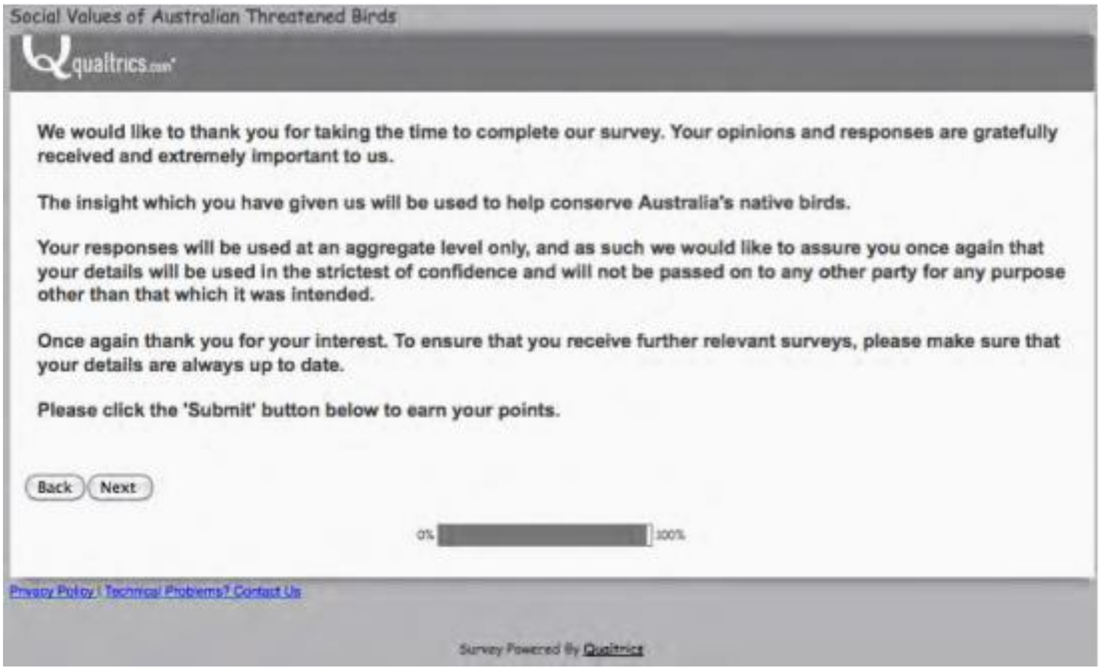
Social Values of Australian Threatened Birds

qualtrics.com

We would like to thank you for taking the time to complete our survey. Your opinions and responses are gratefully received and extremely important to us.

The insight which you have given us will be used to help conserve Australia's native birds.

Your responses will be used at an aggregate level only, and as such we would like to assure you once again that your details will be used in the strictest of confidence and will not be passed on to any other party for any purpose other than that which it was intended.

Once again thank you for your interest. To ensure that you receive further relevant surveys, please make sure that your details are always up to date.

Please click the 'Submit' button below to earn your points.

Back Next

0% 100%

Privacy Policy | Technical Problems? Contact Us

Survey Powered By Qualtrics
