# Supplementary figures and images for: Are we choosing the right flagships? The bird species and traits Australians find most attractive
Source: PLoS One. 2018 Jun 26;13(6):e0199253. doi: 10.1371/journal.pone.0199253 (PMC6019765; doi:10.1371/journal.pone.0199253)

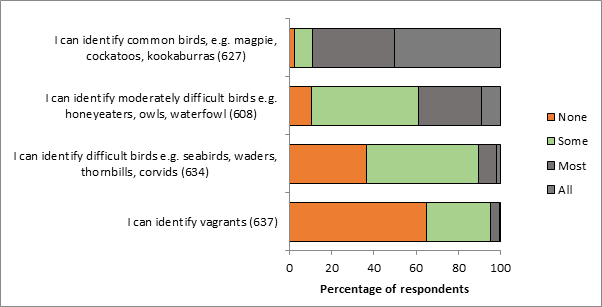

Supplement: S1 Fig — (TIF) [file pone.0199253.s002.tif]

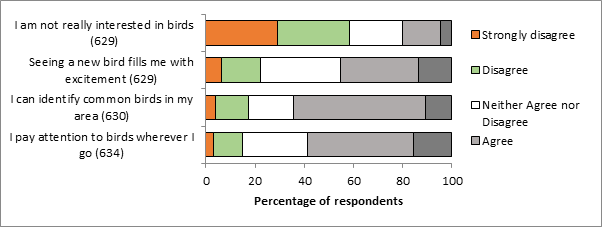

Supplement: S2 Fig — (TIF) [file pone.0199253.s003.tif]
